# 3D hierarchically gold-nanoparticle-decorated porous carbon for high-performance supercapacitors

**DOI:** 10.1038/s41598-019-53506-6

**Published:** 2019-11-19

**Authors:** Hongfang Ma, Zhanghao Chen, Xiang Gao, Wenfei Liu, Hanfei Zhu

**Affiliations:** 1 School of Environmental Science and Engineering, Qilu University of Technology (Shandong Academy of Sciences), Jinan, 250353 China; 2 School of Materials Science and Engineering, Shandong Jianzhu University, Jinan, 250101 China; 3 Institute of Advanced Energy Materials and Chemistry, School of Chemistry and Pharmaceutical Engineering, Qilu University of Technology (Shandong Academy of Sciences), Jinan, 250353 China; 4 Department of Chemistry and Biochemistry, University of California, Los Angeles, Los Angeles, California, 90095 United States

**Keywords:** Supercapacitors, Nanoscale materials

## Abstract

Porous carbon are excellent electrode materials for energy-storage devices. Here, we present a facile *in-situ* reduction method to improve the electrochemical performance of carbon materials by gold nanoparticles. The prepared porous carbon microspheres decorated with gold-nanoparticle had a 3D honeycomb-like structure with a high specific surface area of about 1635 m^2^ g^−1^, confirmed by scanning electron microscopy, transmission electron microscopy, and the Brunauer-Emmett-Teller method. The electrochemical performance of as-synthesized porous carbon microspheres was exemplified as electrode materials for supercapacitor with a high specific capacitance of 440 F g^−1^ at a current density of 0.5 A g^−1^, and excellent cycling stability with a capacitance retention of 100% after 2000 cycles at 10 A g^−1^ in 6 M KOH electrolyte. Our method opened a new direction for the gold-nanoparticle-decorated synthesis of porous carbon microspheres and could be further applied to synthesize porous carbon microspheres with various nanoparticle decorations for numerous applications as energy storage devices, enhanced absorption materials, and catalytical sites.

## Introduction

The exploitation and utilization of sustainable energy sources such as solar, wind, and tidal energies are receiving extensive research attention because of the overconsumption of traditional fossil energy and environmental deterioration^1,2^. Meanwhile, research efforts into the design and development of advanced devices for energy conversion and storage are essential^3^. Supercapacitor, also known as electrochemical capacitor, is widely used in electronics and hybrid vehicles as a new type of promising green energy storage device due to high power density and long cycle stability^4–8^. As one of the critical factors determining the performance of supercapacitors, electrode materials are extensively studied. Carbon-based materials such as porous carbon, carbon nanotubes, carbon nanofibers, and graphene, are widely studied for supercapacitors application owing to their high specific surface area, excellent chemical stability, and environmental compatibility^9–13^. However, preparations of some carbon-based materials are cumbersome, resulting in high cost and low yield. Thus, effectively preparing carbon materials from waste biomass and designing into energy storage devices become attractive.

Biomass resources are abundant in the world, so carbon materials prepared from waste biomass are eco-friendly and low cost, which are crucial to environmental protection and sustainable development. Thus, numerous studies focus on utilizing waste biomass such as rice husk, cauliflower leaf, and almond shell, to produce carbon materials for supercapacitor electrodes^14–16^. Generally, high specific surface area of carbon materials often leads to high capacitance, to further increase the specific surface area and electrochemical performance, researchers are keen to make carbon-based materials with various shapes or porous structures, but the conductivity of carbon materials is also sacrificed. Traditionally, graphene has been doped into active materials to improve their conductivity^17–19^. However, graphene is expensive to produce, and the graphite from mineral and petroleum as primary raw materials preparing graphene is very limited, which is not conducive to sustainable development. Therefore, it is critical to find an alternative way replacing graphene doping.

Gold is a highly conductive material, and gold nanoparticles possess excellent stability, surface effects, and various catalytic properties. It is widely used in sensing^20^, drug delivery^21^, and catalysis^22^. Recently, gold nanoparticles have been combined with electrode materials to enhance conductivity. For example, Chaudhari and co-workers prepared Au-MWCNT composites, which showed lower Au loading displayed higher specific capacitance of 105 F g^−1^, demonstrating that decorated by gold nanoparticles with the smaller particle is more effective in enhancing the capacitance of the composite^23^. Jiang *et al*. synthesized Au@nitrogen-doped carbon nanocage, which displayed cycling stability with 97% of capacitance retained after 5000 cycles^24^. The core-shell nano-Au@PANI was prepared by Tan *et al*. *via in-situ* polymerized polyaniline. Electrochemical tests show that this material has the highest specific capacitance reaching 285 F g^−1^ ^25^. The high conductivity, good stability, and no faradic effect make gold nanoparticles possible to be used in electrical double layer capacitors (EDLCs). However, few studies have reported the synthesis of porous carbon decorated with gold nanoparticles for the application in EDLCs.

**Figure 1:**
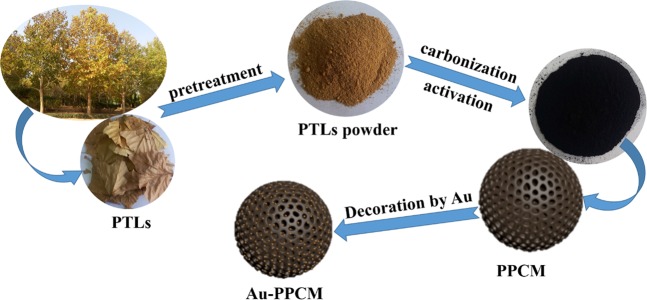
Schematic of Au-PPCM preparation process.

In this work, porous carbon microspheres were prepared from fallen phoenix tree leaves (PTLs), and decorated with thimbleful gold nanoparticles. Fallen PTLs are everywhere in north China in autumn, but most of them are burned, causing resource wastage and exacerbated air pollution. Thus, it is environment-friendly and sustainable to employ renewable and widespread PTLs as raw materials to prepare PTL-based porous carbon microspheres (PPCMs). Fallen PTLs were treated through hydrothermal carbonization and chemical activation to obtain PPCM. Then a small amount of gold nanoparticles were decorated on the prepared PPCM (Au-PPCM) *via* a simple *in-situ* reduction method. The general process of synthesizing Au-PPCM is illustrated in Fig. 1. The resulting Au-PPCM composites had gold nanoparticles in uniform size and good dispersibility. The obtained PPCM and Au-PPCM were characterized and evaluated as the electrode material. And the mechanism of gold nanoparticles enhancing electrochemical properties of PPCM was also discussed.

## Results

**Figure 2:**
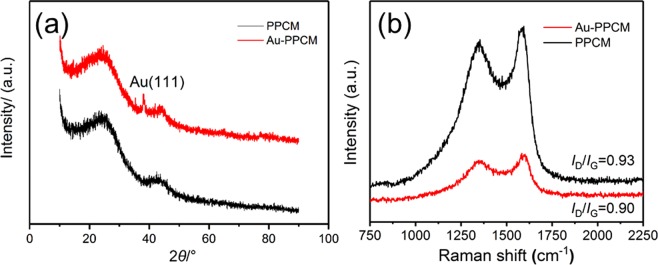
(**a**) XRD and (**b**) Raman patterns of PPCM and Au-PPCM.

From the XRD patterns (Fig. 2a), both PPCM and Au-PPCM composites had two broadened diffraction peaks at 22° and 43°, corresponding to the (002) and (101) reflection of graphite stacking^26–28^, indicating that the prepared carbon materials had a certain degree of graphitization. While the other characteristic diffraction peak of Au-PPCM emerged at 38.4°, which was ascribed to the (111) crystal plane of gold^29–31^, illustrating that gold nanoparticles were successfully decorated on PPCM. Meanwhile, two evident bands were observed in the Raman spectra of all the samples at approximately 1350 and 1750 cm^−1^, which represent the defect (D) and graphitic (G) bands, respectively (Fig. 2b). The ID/IG ratios were calculated to be 0.93 and 0.90 for PPCM and Au-PPCM, respectively. These results demonstrated that the samples were partial graphitization.

**Figure 3:**
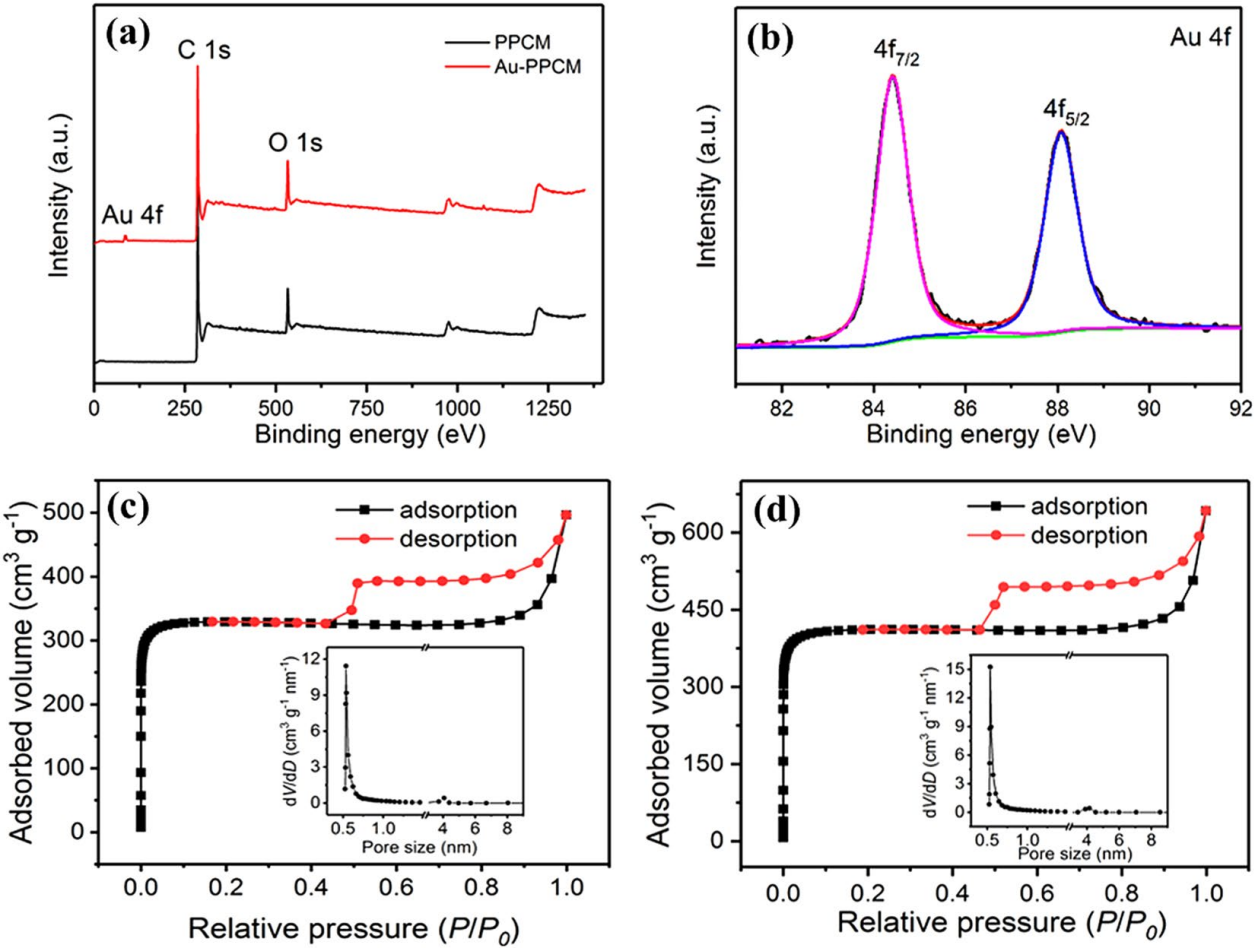
(**a**) XPS spectrum, (**b**) Au 4 f XPS spectrum of Au-PPCM, and N_2_ adsorption–desorption isotherms of (**c**) PPCM and (**d**) Au-PPCM. The inset shows the corresponding pore-size distributions.

XPS measurement was performed to further confirm the composition of the samples, as shown in Fig 3a,b. The survey-scan XPS spectra (Fig. 3a) of Au-PMCC exhibited the characteristic peaks of C 1 s, O 1 s, and Au 4 f, whereas PMCC evaluated only C 1 s and O 1 s. Moreover, the high-resolution XPS spectra of Au 4 f for Au-PMCC (Fig. 3b) could be better fitted with a couple of doublet peaks with binding energies at 84.0 and 87.9 eV, corresponding to Au 4f_7/2_ and Au 4f_5/2_^32,33^ respectively. These findings further confirmed the existence of gold nanoparticles on PPCM. The N_2_ adsorption/desorption isotherms of PPCM and Au-PPCM were obtained, and results are depicted in Fig. 3c,d (the inset shows the corresponding pore-size distributions). Both PPCM and Au-PPCM exhibited a type-IV isotherm. The curves sharply rose at low relative pressure, demonstrating the existence of micropores. Meanwhile, typical hysteresis loops can be found at medium relative pressure, indicating the presence of mesopores. Notably, both PPCM and Au-PPCM had considerable micropores with the size of about 0.54 nm and some mesopores. The same pore-size distribution demonstrated that gold nanoparticles were not doped into the micropores of PPCM to block the micropores channel but adsorbed on its surface or the inner surface of mesopores. This structure was beneficial to increase the specific surface area and ion transport rate. The BET specific surface area of PPCM was 1294 m^2^ g^−1^, which was smaller than that of Au-PPCM (1635 m^2^ g^−1^). The reason was that gold nanoparticles on PPCM formed a micro/nano-structure so that the specific surface area of Au-PPCM became larger. The higher specific surface area of Au-PPCM resulted in larger number of active sites. Thus, numerous charges can be arranged during the electrochemical processes^24^.

**Figure 4:**
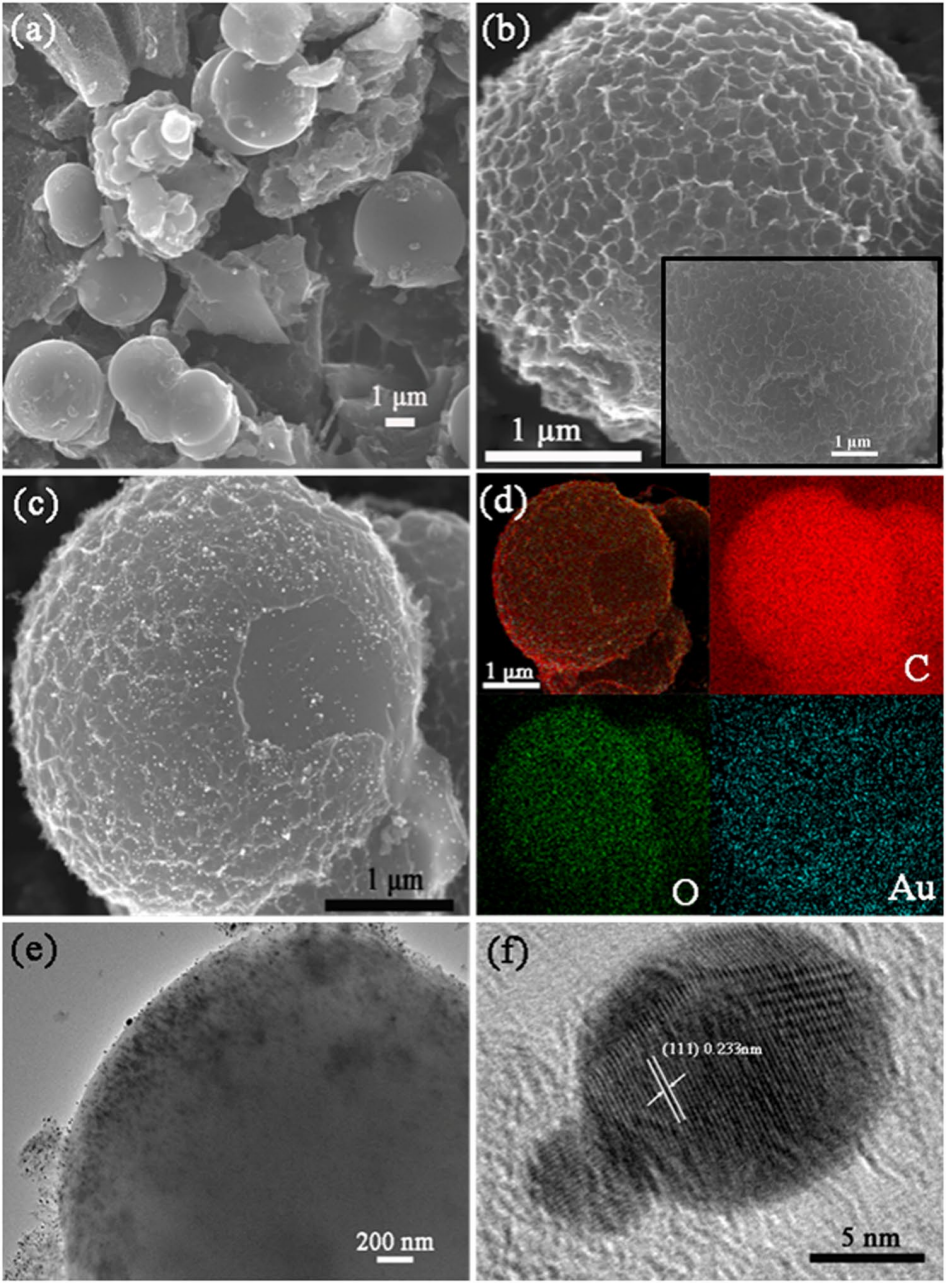
SEM image of (**a**,**b**) PPCM and (**c**) Au-PPCM, (**d**) corresponding elemental mapping of Au-PPCM, (**e**) TEM image of Au-PPCM, and (**f**) HRTEM image of Au nanoparticles of Au-PPCM.

Figure 4a,b show the SEM images of the as-prepared PPCM. Notably, PPCM possessed the characteristic three-dimensional carbon microsphere architectures. At higher magnification, another exciting feature was present. Abundant pores were evenly distributed on the carbon microsphere, forming a 3D honeycomb-like porous structure. This unique structure ensued owing to hydrothermal carbonization and KOH activation. The hydrothermal carbonization led to the formation of carbon microspheres, and the final products showed a honeycomb-like porous morphology after KOH activation. According to previous reports, we attributed the formation of this porous feature to the reaction of KOH and carbon materials^34^. The finally porous structure formed possibly by the degassing process of carbon dioxide and some carbon monoxide. The particular structure increased the specific surface area of the carbon material, which was helpful to accumulate more charges and electrolyte ions and can facilitate the formation of the electric double layer, thereby improving the electrochemical performance of the carbon material. Furthermore, this structure also provided more adsorption sites for chloroauric acid, which was beneficial for the *in-situ* reduction of gold nanoparticles on carbon microsphere. As shown in Fig. 4c, Au-PPCM also had three-dimensional honeycomb-like architectures, indicating that the *in-situ* reduction of gold nanoparticles did not change the original structure of PPCM. The corresponding elemental mapping of Au-PPCM demonstrated that Au-PPCM contained three elements, C, O, and Au, which was consistent with the results of XPS. In combination with Fig. 4c, the gold nanoparticles can be seen to be well dispersed on the PPCM surface. This was owing to the original honeycomb-like structure providing more and more uniform adsorption sites for gold nanoparticles.

The structural details of Au-PPCM were further examined by TEM and HRTEM. Figure 4e shows the TEM image of Au-PPCM, which confirmed that the distributed gold nanoparticles uniformly adsorbed on the surface of PPCM as the images SEM showed. The typical HRTEM image of Au nanoparticles was exhibited in Fig. 4f and the obtained Au nanoparticles by *in-situ* reduction of sodium borohydride had particle size of about 10 nm. Moreover, the HRTEM image of gold nanoparticles showed that the lattice fringe of these gold nanoparticles had an interlayer distance of 0.233 nm, which can be attributed to the (111) crystal plane of metal gold crystals^35,36^ and agreed with the XRD analysis of Au-PPCM.

**Figure 5:**
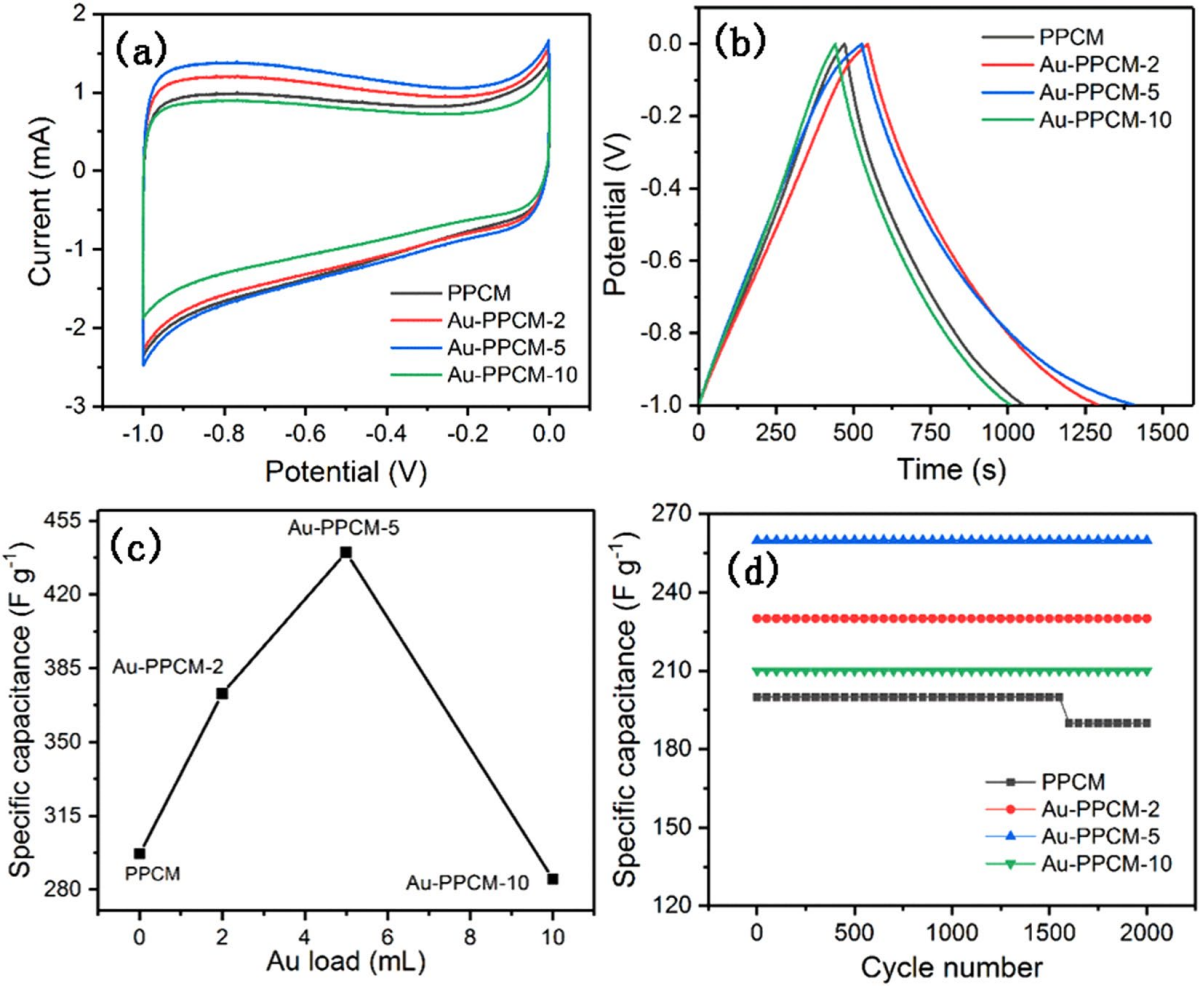
(**a**) CV curves of PPCM and Au-PPCM at different Au loadings at a scan rate of 5 mV s^−1^, (**b**) GCD curves of PPCM and Au-PPCM at different Au loadings at a current density of 0.5 A g^−1^, (**c**) specific capacitances of PPCM and Au-PPCM at different Au loading at a current density of 0.5 A g^−1^, (**d**) Cyclic performance of PPCM and Au-PPCM at different Au loadings.

**Figure 6:**
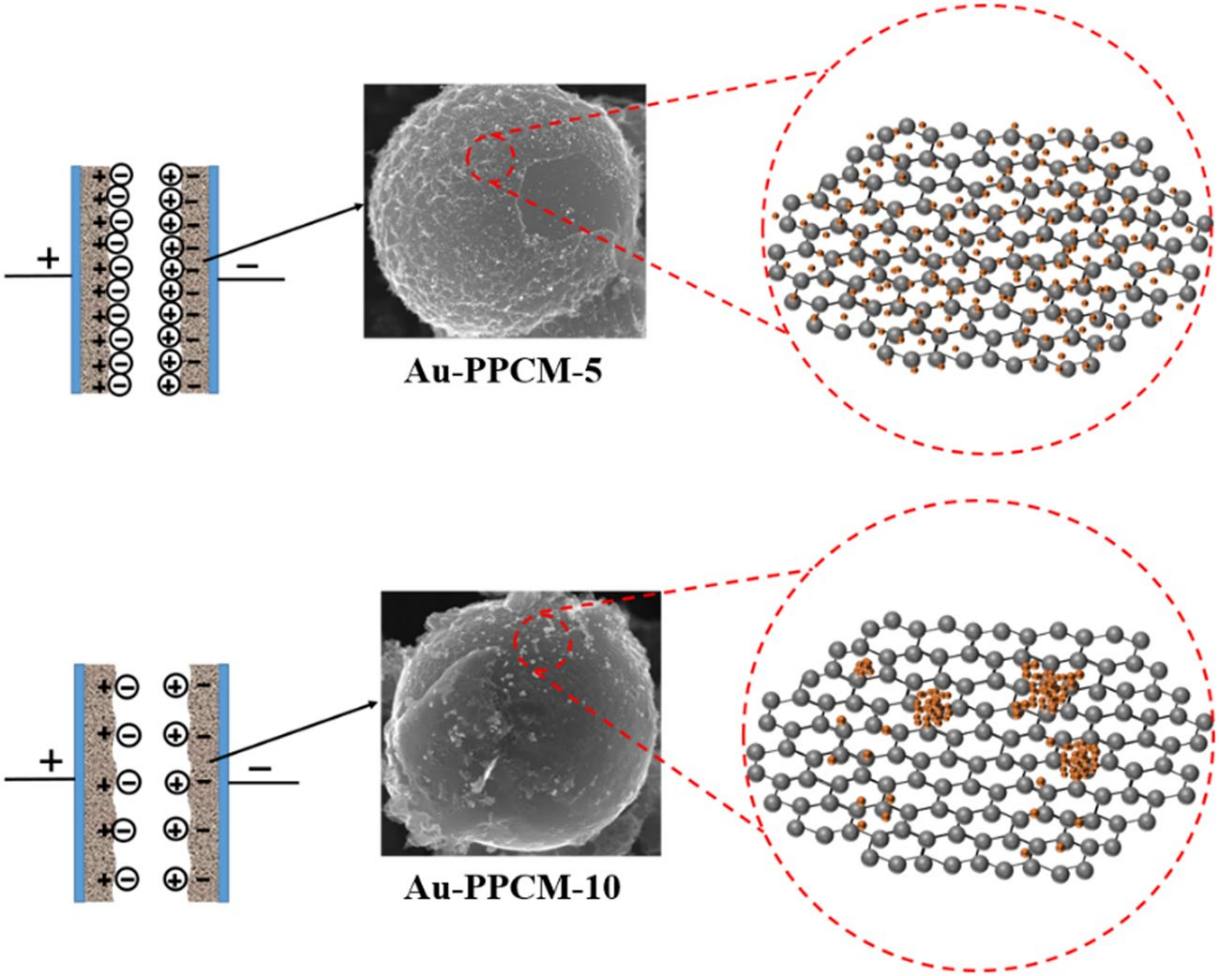
Schematic of double electric layers with different Au loadings of Au-PPCM.

The electrochemical performance of as-prepared PPCM and Au-PPCM were investigated by CV, GCD, and EIS using a three-electrode system in 6 M KOH electrolyte. Figure 5a shows the CV curves of PPCM and Au-PPCM with different Au loadings. Both PPCM and Au-PPCM exhibited characteristic quasi-rectangular curves without any redox peaks, indicating excellent electric double-layer capacitive characteristics^37^. The area enclosed by CV curves can reflect the value of specific capacitance^25^. Compared with the other three materials, Au-PPCM-5 showed enhanced specific capacitance that can be further confirmed by GCD performance. The GCD curves of the electrodes are shown in Fig. 5b. All GCD curves exhibited a typical quasi-triangular symmetrical distribution, indicating that the electrode materials had good reversibility, which was also typical of electric double-layer capacitive characteristics^38^. According to GCD test results, the specific capacitance of electrodes can be calculated by Eq. (1), as shown in Fig. 5c. At a current density of 0.5 A g^−1^, the specific capacitances of PPCM, Au-PPCM-2, Au-PPCM-5, and Au-PPCM-10 were 297, 373, 440, and 285 F g^−1^, respectively. These results well agreed with the CV findings. The low gold loading of Au-PPCM exhibited a higher specific capacitance, whereas the high gold loading of Au-PPCM-10 had a lower specific capacitance even compared with PPCM. According to a previous report^23^, we speculated that the low gold loading of Au-PPCM could accumulate more charges, thereby increasing the specific capacitance. However, the higher Au loading of Au-PPCM-10 provided a fast conduction path for electron transfer, resulting in lower charge accumulation and specific capacitance. Another explanation was that higher Au loading could lead to the conglomeration of gold nanoparticles on the surface of PPCM and in turn clog the pores of the carbon materials resulting in a reduction in specific surface areas (1635 m^2^ g^−1^ for Au-PPCM-5 and 1401 m^2^ g^−1^ for Au-PPCM-10), and then can accumulate less charges, further showing lower specific capacitance. The mechanism diagram is illustrated in Fig. 6. In addition, the specific capacitances of different carbon materials used in literature and in this study were listed in Table 1.The results indicated that performance of Au-PPCM is comparable or higher than that of other similar materials.

**Table 1:** Comparison of specific capacitance between difference carbon sources.

Materials	Specific capacitance(F·g^−1^)	Cell remarks	Refs
rice husk	367	three electrodes	^14^
ginkgo shells	178	three electrodes	^37^
bagasse	320	three electrodes	^26^
Au-MWCNT	105	two electrodes	^23^
Au@NCNC	168.6	three electrodes	^24^
Au-PPCM	440	three electrodes	This work

Cycle performance of the electrode is also an important indicator to measure electrode quality. To further examine the cycling stability of the electrodes, constant current charge/discharge cycling at a current density of 10 A g^−1^ was evaluated (Fig. 5d). After 2000 cycles, the specific capacitance of Au-PPCM (Au-PPCM-2, Au-PPCM-5, and Au-PPCM-10) almost remained the same without attenuation, whereas PPCM was attenuated by 5% of its initial capacitance. The reason was that Au nanoparticles can provide the necessary electric-transport channel during the charge/discharge process, avoiding the electrode attenuating^25^.

**Figure 7:**
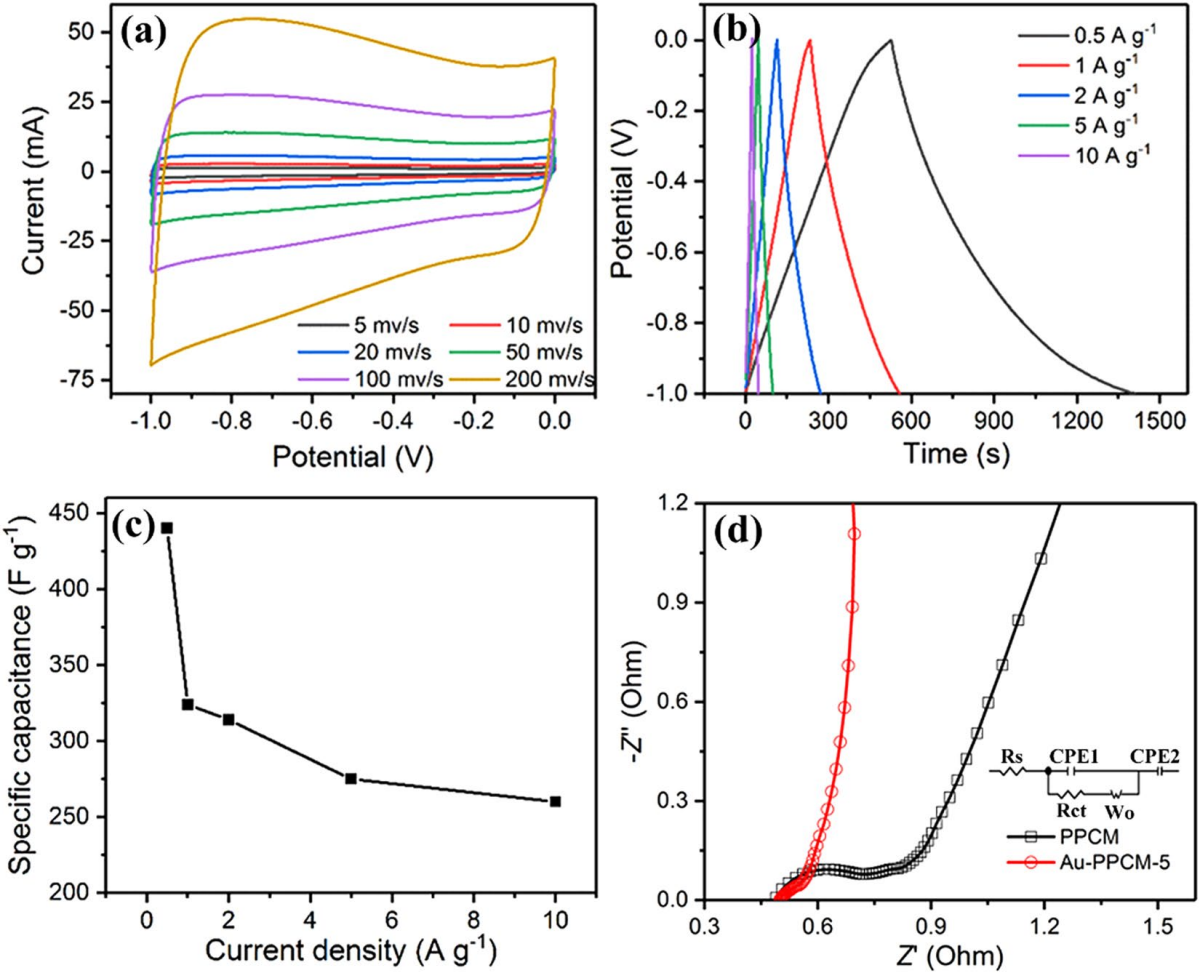
Electrochemical performance of the Au-PPCM-5: (**a**) CV curves at different scan rates, (**b**) GCD curves at different current densities, (**c**) specific capacitances at different current densities, (**d**) EIS Nyquist plots for PPCM and Au-PPCM-5.

The electrochemical performance of Au-PPCM-5-based electrode was further measured in 6 M KOH electrolyte, and results are shown in Fig. 7. The CV and GCD curves of Au-PPCM-5 maintained quasi-rectangular and quasi-triangular symmetries even at higher scan rates or current densities, indicating that the capacitance originated almost from the electric double-layer capacitance behavior. The particular 3D honeycomb-like structure with the large accessible specific surface area can significantly enhance the specific capacitance. And the gold nanoparticles decoration can improve the conductivity of the active materials by shortening the ions/charges pathways. The two reasons together caused the CV and GCD curves to maintain a better shape. With increased current density, the specific capacitance of Au-PPCM-5 gradually decreased due to the increased transfer speed of the electrolyte ions/charges, resulting in decreased specific capacitance. Notably, the specific capacitances of Au-PPCM-5 decreased from 324 F g^−1^ to 260 F g^−1^, showing excellent rate performance at high current densities (1 A g^−1^ to 10 A g^−1^). This finding was due to the porous structure and gold nanoparticles facilitating ions/charges transfer and diffusion during the electrochemical process. Furthermore, the specific capacitance value was also calculated from the CV curve using the Eq. (5) as Table 2. The EIS plots of the electrodes are shown in Fig. 7d. The intercept of the high-frequency region and the real axis represents the equivalent series resistance, which is consisted of the inherent resistance of the electrolyte, current collector, and contact resistance^39,40^. The diameter of the arc indicated the charge-transfer resistance (*R*ct), which affected the transfer of ions/charges on the surface of active materials. By electrochemical fitting, the Au-PPCM-5-based electrode exhibited an ultrasmall *R*ct of 0.06 Ω, smaller than the *R*ct (0.39 Ω) of PPCM. This result indicated that the decoration by gold nanoparticles enhanced the conductivity of the electrode material.

**Table 2:** Specific capacitances of Au-PPCM-5 at different scan rates.

Scan rates (mV·s^−1^)	5	10	20	50	100	200
C_S_ (F·g^−1^)	392.34	383.83	379.68	365.15	352.26	333.75

After been modification by gold nanoparticles, the conductivity of carbon was improved and could provide more active sites for charge accumulation which promoted the diffusion and transfer of electrolyte particles and charges. So the electrochemical interaction between the electrolyte and the carbon material are more available and improve the electrochemical properties. On the other hand, gold nanoparticles can form a micro-nano structure with porous carbon materials to increase the specific surface area and maintain the original porous structures which do not block the interaction between the electrolyte particles and the carbon material.

**Figure 8:**
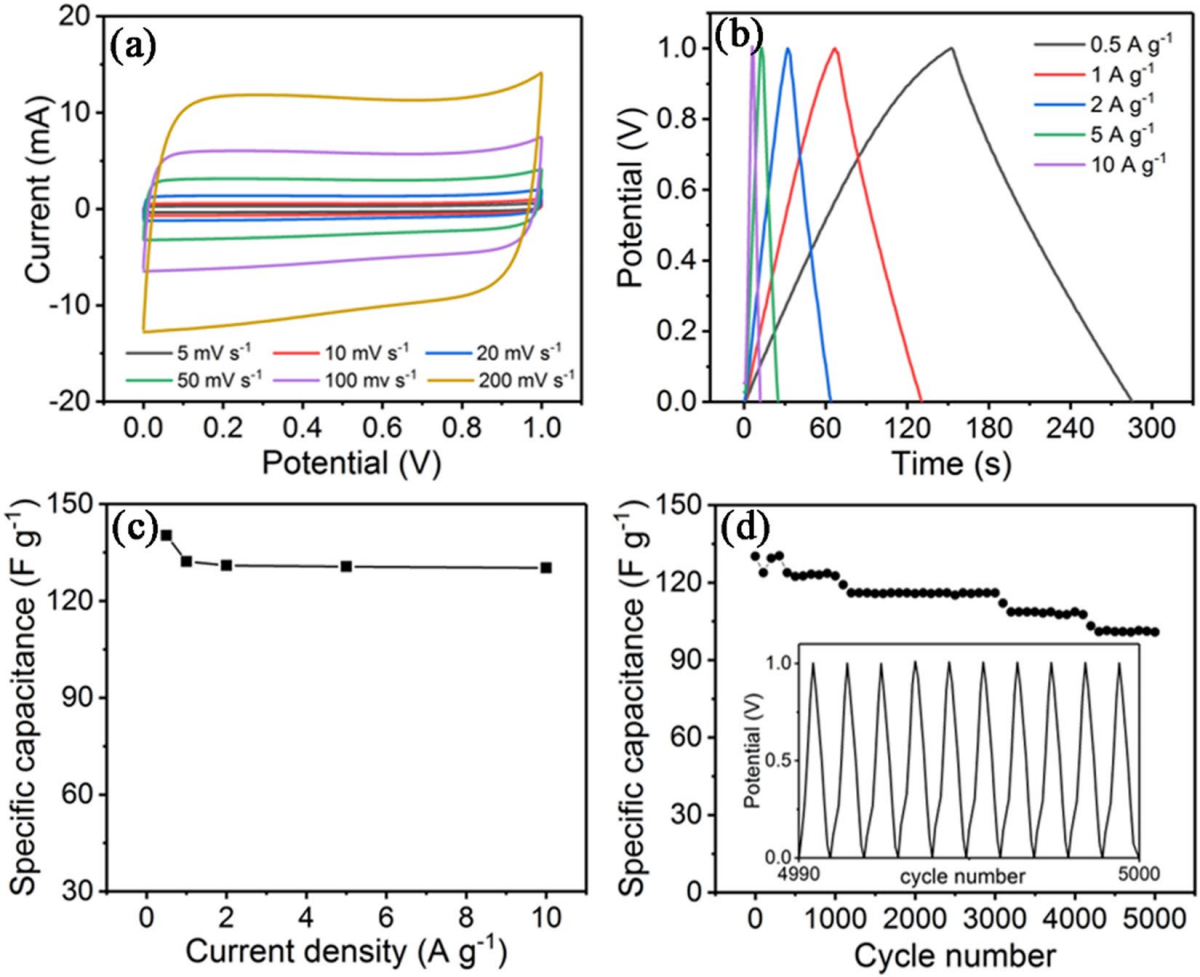
Electrochemical performance of the symmetric supercapacitor: (**a**) CV curves at different scan rates, (**b**) GCD curves at different current densities, (**c**) specific capacitances at different current densities, (**d**) Cycling performance over 5000 cycles at 10 A g^−1^.

The electrochemical performance of symmetric supercapacitor using two as-prepared Au-PPCM-5 electrodes was also tested in a two-electrode system in 6 M KOH electrolyte. Figure 8a shows the CV curves covering the scanning rates ranging from 5 to 200 mV s^−1^. And Fig. 8b shows GCD curves covering the current density ranging from 0.5 to 10 A g^−1^ in the potential range between 0 and 1.0 V. The CV curves exhibited a similar rectangular shape at different scan rates ranging, and all the charge/discharge curves showed typical triangular shape, implying excellent rate ability and electrochemical reversibility. Figure 8c shows a series of specific capacitance values according to Eq. (2). The symmetric electrodes showed the high specific capacitance values of 140.3, 132.2, 131, 130.6 and 130.2 F·g^−1^ at the current density of 0.5, 1, 2, 5 and 10 A·g^−1^, respectively. Even at a higher current density (10 A·g^−1^), it remained up to 130.2 F·g^−1^, which showed an excellent rate performance with 92.8% capacitance retention. The energy density and power density were further calculated according to Eqs (3) and (4). This symmetric supercapacitor could deliver a maximum energy density of 19.47 Wh kg^−1^ at a power density of 499.86 W kg^−1^. At a current density of 10 A g^−1^, the power density increased to 9998.16 W kg^−1^, maintaining the energy density of 18.08 Wh kg^−1^. The cyclic performance of this symmetric supercapacitor was carried out by galvanostatic charge/discharge at a constant current density of 10 A g^−1^ for 5000 cycles, as shown in Fig. 8d. After 5000 cycles, the symmetric supercapacitor retained 77.4% of its initial capacitance, indicating excellent cyclic stability. These results demonstrated that the gold-nanoparticle-decorated porous carbon microspheres have satisfactory electrochemical stability for supercapacitor application.

## Conclusions

Novel 3D honeycomb-like gold-nanoparticle-decorated porous carbon microspheres were successfully synthesized *via* a simple and effective *in-situ* reduction method. The prepared composites with different Au loadings were applied as electrode materials. Compared with PPCM, Au-PPCM-5 exhibited the best electrochemical performance, which had larger specific capacitance, better cycle performance, and higher electrical conductivity. The Au-PPCM-5 electrode has the preeminent specific capacitance, which is up to 440 F g^−1^ at a current density of 0.5 A g^−1^ and shown excellent stability (the specific capacitance did not attenuate even after 2000 cycles). Moreover, the composite also demonstrated a higher energy density of 19.47 Wh kg^−1^ and a power density of 499.86 W kg^−1^ at a current density of 0.5 A g^−1^. The approach provided an excellent way to use sustainable resources from waste biomass and a novel idea for increasing the specific capacitance of materials in the energy field, which has high potential applications in energy storage devices.

## Methods

### Synthesis of Au-PPCM

Based on our previous report^41^, PPCM was prepared through a two-step process. First, carbon microspheres were prepared from PTLs *via* hydrothermal carbonization at 220 °C for 12 h. The porous structure was formed through chemical activation with potassium hydroxide under nitrogen flow (The temperature and holding time were set as 450 °C for 30 min, 650 °C for 30 min, and 800 °C for 60 min). Au-PPCM composites with different Au loadings were prepared. To synthesize Au-PPCM with 5 mL of chloroauric acid (denoted as Au-PPCM-5), 5 mL of 1 mM chloroauric acid was added into a centrifuge tube and diluted to 20 mL. Then, 0.1 g of the prepared PPCM was added to the solution under stirring at 180 r/min for 3 h. Afterward, 2 mL of freshly prepared 4 mM sodium borohydride solution was quickly added under magnetic stirring for 30 min. The reaction products were centrifuged and washed with distilled water and anhydrous ethanol, and then dried to obtain Au-PPCM-5 samples. Similar procedures were followed to prepare Au-PPCM composites with 2 and 10 mL of chloroauric acid (denoted as Au-PPCM-2 and Au-PPCM-10, respectively).

### Materials characterization

Crystal structures were analyzed through X-ray diffraction (XRD; D8 Bruker) with Cu Kα radiation. X-ray photoelectron spectroscopy (XPS) was performed on an Escalab 250Xi (Thermo Scientific, Britain) system with Al Kα excitation. Morphologies were examined by scanning electron microscopy (SEM; Regulus8220, Japan). Transmission electron microscopy (TEM; JEM-2100, JEOL, Japan) was used to collect TEM and high-resolution TEM (HRTEM) images. Nitrogen adsorption/desorption isotherms were obtained at 77 K using a KUBO-X1000 (Builder Electronic, China). The specific surface area was calculated by the Brunauer-Emmette-Teller (BET) method, and pore-size distributions were evaluated by the Horvath-Kawazoe (HK) model for micropores and Barrett-Joyner-Halenda model for mesopores.

### Electrochemical measurements

The electrochemical performance was measured in 6 M potassium hydroxide solution using a three-electrode system. This system included the as-prepared active materials as the working electrode, a platinum foil electrode as the counter electrode, and a saturated calomel electrode as the reference electrode. The working electrode was prepared as follows: the obtained active materials were mixed with acetylene black and polyvinylidene fluoride at 8:1:1 weight ratio along with a few drops of N-methylpyrrolidone solution under stirring until a homogeneous slurry formed. Then the slurry was coated onto nickel mesh about 1 cm^2^ (active material loading was about 1–2 mg). Finally, the as-prepared electrodes were dried at 80 °C for 8 h and pressed at 20 MPa for 5 min.

Cyclic voltammetry (CV), electrochemical impedance spectroscopy (EIS), and galvanostatic charging/discharging (GCD) measurements were used to characterize the electrochemical performance of the electrodes. CV and EIS were performed using a CHI660E electrochemical workstation. CV analysis was carried out between −1 V to 0 V at different scan rates ranging from 5 mV s^−1^ to 200 mV s^−1^, and EIS was performed at a *V*_oc_ with an amplitude of 5 mV and within the frequency range of 0.01 Hz to 100 kHz. GCD measurements were conducted on a CT2001A land battery measurement system at test voltages of −1 V to 0 V and different current densities from 0.5 A g^−1^ to 10 A g^−1^. The specific capacitance was calculated from GCD according to Eq. (1):

1 $$C=I\Delta t/(\Delta Vm)$$

where *C* is the specific capacitance (F g^−1^), *I* is the discharge current (A), Δ*t* is the discharge time (s), *m* is the active material mass on the electrode material (g), and Δ*V* is the test potential window (V).

Towards practical use, a symmetric supercapacitor using two Au-PPCM electrodes was measured in a two-electrode system in 6 M KOH. The specific capacitance of the supercapacitor is calculated according to Eq. (2):

2 $$C=2I\Delta t/(\Delta Vm)$$

The energy density (*E*, Wh kg^−1^) and power density (*P*, W kg^−1^) of the symmetric supercapacitor can be calculated based on the following Eqs (3) and (4):

3 $$E=C\Delta {V}^{2}/7.2$$

4 $$P=3600\,E/\Delta t$$

The specific capacitance values calculated from the CV curves use the Eq. (5):

5 $$C=\frac{1}{2{ms}({V}_{f}-{V}_{i})}{\int }_{{V}_{i}}^{{V}_{f}}I(V)d{V}$$

where *m* is the mass of the active electrode material, *s* is the scan rate, *V*_*f*_ and *V*_*i*_ are the integration limits of the voltammetric curve, and *I(V)* denotes the current response, respectively.
